# Cancer patients spend more time at home and more often die at home with advance care planning conversations in primary health care: a retrospective observational cohort study

**DOI:** 10.1186/s12904-022-00952-1

**Published:** 2022-05-02

**Authors:** Bardo Driller, Bente Talseth-Palmer, Torstein Hole, Kjell Erik Strømskag, Anne-Tove Brenne

**Affiliations:** 1 Department of Oncology and Department for Research and Innovation, Møre and Romsdal Hospital Trust, Åsehaugen 1, N-6026 Ålesund, Norway; 2 Department of Clinical and Molecular Medicine, Faculty of Medicine and Health Sciences, Norwegian University of Science and Technology, Trondheim, Norway; 3 School of Biomedical Sciences and Pharmacy, College of Health, Medicine and Wellbeing, University of Newcastle, Australia and NSW Health Pathology, New South Wales, Australia; 4 Operating department, Møre and Romsdal Hospital Trust, Ålesund, Norway; 5 Department of Circulation and Medical Imaging, Faculty of Medicine and Health Science, Norwegian University of Science and Technology, Trondheim, Norway; 6 Department of Surgery and Emergency Medicine Molde Hospital, Møre and Romsdal Hospital Trust, Molde, Norway; 7 Cancer Clinic, St. Olavs Hospital, Trondheim University Hospital, Trondheim, Norway

**Keywords:** Advance Care Planning, Cancer, Palliative Care, Primary Health Care, Home Care, Place of death, Home death

## Abstract

**Background:**

Spending time at home and dying at home is advocated to be a desirable outcome in palliative care (PC). In Norway, home deaths among cancer patients are rare compared to other European countries. Advance care planning (ACP) conversations enable patients to define goals and preferences, reflecting a person’s wishes and current medical condition.

**Method:**

The study included 250 cancer patients in the Romsdal region with or without an ACP conversation in primary health care who died between September 2018 and August 2020. The patients were identified through their contact with the local hospital, cancer outpatient clinic or hospital-based PC team.

**Results:**

During the last 90 days of life, patients who had an ACP conversation in primary health care (*N*=125) were mean 9.8 more days at home, 4.5 less days in nursing home and 5.3 less days in hospital. Having an ACP conversation in primary health care, being male or having a lower age significantly predicted more days at home at the end of life (*p*< .001). Patients with an ACP conversation in primary health care where significantly more likely to die at home (*p*< .001) with a four times higher probability (RR=4.5). Contact with the hospital-based PC team was not associated with more days at home or death at home. Patients with contact with the hospital-based PC team were more likely to have an ACP conversation in primary health care.

**Conclusion:**

Palliative cancer patients with an ACP conversation in primary health care spent more days at home and more frequently died at home. Data suggest it is important that ACP conversations are conducted in primary health care setting.

**Supplementary Information:**

The online version contains supplementary material available at 10.1186/s12904-022-00952-1.

## Introduction

A diagnosis of non-curable cancer challenges us to think about, talk about and plan for future health. Advance care planning (ACP) enables patients to define goals, preferences for future medical treatment and care, to discuss these goals and preferences with family and health care providers, and to record and review these preferences if appropriate [1]. Building on a well-established patient relationship and mutual trust, the general practitioner (GP) and primary health care nurse can make the necessary ACP conversation meaningful [2, 3]. In real life, ACP is often performed in the terminal phase, discussed but not documented or not considered at all [4]. The right time and place for an ACP conversation is an ongoing discussion between specialist and primary health care [5, 6]. The conversation is easier with a patient that is not suffering from serious symptoms, and it might be more meaningful at home or the place where the patient wants to be during final weeks/months of life.

With an increasing symptom burden and disability, transitions between different care facilities can be challenging and the necessary treatment is more fragmented [7]. Limited hospital-based specialist palliative care (PC) resources meet a raising number of palliative cancer patients, living longer with modern oncology treatment [8]. During End-of-Life (EoL) care these patients often experience insufficient assessment and documentation of their goals of care, which contributes to repeated and sometimes burdensome admissions to hospital [9, 10]. Patients and family caregivers want health professionals to work collaboratively. A possible way for future strategy could be a joint responsibility, where a hospital-based program prepares patients and relatives for the transition to a home-based program with a primarily responsible GP [11].

Dying at home or dying in the preferred place is advocated as an outcome of high-quality PC, time at home at the EoL may be an even more important quality indicator [12, 13]. Patients with cancer who die in a hospital or intensive care unit (ICU) can have worse quality of life (QoL) compared with those who die at home [14]. EoL transitions between health care settings for variable reasons are common across EU countries, in particular late hospitalizations for people residing at home [10]. Cancer deaths occurring at home in 2003 was 12.8% in Norway, 22.1% in England, 22.7% in Wales, 27.9% in Belgium, 35.8% in Italy, and 45.4% in the Netherlands [15]. In a study covering all 83.434 deaths in Norway in 2012 and 2013, 15% of deaths happened at home, most frequent among patients with 'Circulatory diseases' and 'Cancer' [16]. Little is known about where palliative cancer patients in Norway spend their time during the last months of life. EoL cancer care seems to be more hospital-centred in Norway with high expenditures [17]. Several factors, like preferences of the patient and caregiver/family or the availability of a palliative home care service influence preferred as well as actual place of care and death [18, 19]. Asking the patient about their own wishes is essential to target future individual care. Qualitative research shows that home environment enabled normality, a sense of control and individualised care, which family carers often perceived as contributing towards a good death [20].

Between January and June 2018, ACP conversations and a summarizing palliative plan was systematically implemented in primary health care in Møre and Romsdal county in Norway. A strategy around implementation was designed to foster a culture of conversation, planning and documentation of patient preferences for care, life priorities and goals in primary health care.

The current study explores the effect of implementing ACP conversations in primary health care on number of days at home at the EoL and on home deaths for palliative cancer patients. We hypothesized that cancer patients having an ACP conversation in primary healthcare in Møre and Romsdal spent more time at home at the end of life and more frequently died at home.

## Methods

### Setting

Community cancer nurses in Møre and Romsdal in North-western Norway had expertise in PC and offered support to patients and family caregivers in addition to the support from GPs and home-care nurses before, during and after the study period. The participating municipalities collaborated with the local hospital, and all cancer patients had access to the hospital-based PC team on referral from hospital or primary health care. The interdisciplinary PC team at the local hospital comprised specialist nurses, PC physicians, social workers, physiotherapists, home health occupational therapist, nutritionist and a chaplain. The team performed home visits in the communities upon request and provided education and support for the patients and their families. Health care providers in the municipalities had the possibility to contact a hospital PC physician by phone 24/7. There is no hospice in Møre and Romsdal.

In 2018, Møre and Romsdal county started providing organized ACP conversations and a structured palliative plan in primary health care to all individuals with life-limiting illnesses like non-curable cancer. From January to June 2018, nurses and physicians in primary health care were trained in planning and organizing ACP conversations and documenting conclusions as a palliative plan. A standardized template for palliative plan was made available in the electronic patient journal (EPJ), an ACP conversation guide and an information video was published on the related website *www.palliativplan.no*. Physicians from the hospital-based PC team informed GPs through visits at the GP offices and through training courses within general PC. Community cancer nurses were trained during routine meetings within the established PC network and were responsible to spread information among home care nurses in their community. Information flyers for health care providers and patients were distributed and helped to prepare and support ACP conversations and a structured palliative plan. Implementation procedures described how to summarize and document the palliative plan in the EPJ, including the possibility to send the plan electronically to any future health care provider in our region. As a major aim of the implementation process, we asked primary health care providers to offer an ACP conversation and a palliative plan to all patients with life-limiting illnesses like non-curable cancer. The local hospital-based PC team recommended ACP conversations and palliative plan in primary health care for all palliative cancer patients in every discharge and outpatient summary.

### Study design

The current study is a retrospective observational cohort study evaluating the place of care prior to death and place of death for patients with incurable cancer who either did or did not undertake an ACP conversation in primary health care settings. When patients had an ACP conversation, conclusions were documented in their EPJ as a structured palliative plan with consent from the patient. The study was conducted in nine municipalities in the Romsdal region with 65.000 inhabitants.

### Subjects

The study included cancer patients who 1) lived in one of the nine municipalities in the Romsdal region, 2) had contact with the local hospital, cancer outpatient clinic or hospital-based PC team, and 3) died between September 2018 and August 2020.

### Patients with ACP conversation in primary health care

After implementation in 2018, patients and/or their relatives had organized ACP conversations together with health care providers to consider patient’s wishes and preferences towards future health care. The primary health care providers received necessary information about medical status and prognosis of the patient from specialist health care. All participants had information about the intention of the ACP conversation. Confirmed conclusions from the ACP conversation were documented as a palliative plan in community EPJ. With permission from the patient, the plan was electronically available for all future health care providers in the region. The palliative plan was reassessed on demand when the patient’s medical condition changed, normally but not always based on a new ACP conversation.

During the implementation process and study period, a general recommendation to offer ACP conversations and palliative plan to all cancer patients treated with palliative intent was the only guidance for primary health care providers in the selection of patients who got an organized ACP conversation. Primary health care providers, mostly community cancer nurses but also home care nurses and GPs, decided if and when the patient should be offered an ACP conversation, and they were responsible for the conduction. They organized the conversation at the patients preferred place, proposing the possibility of having it at home. Patients who received at least one organized ACP conversation in primary health care and had it documented in their medical notes in community EPJ, were in the ACP conversation group.

### Control group

The control group consisted of cancer patients who did not have an ACP conversation and a palliative plan in primary health care setting.

### Primary and secondary outcomes

Primary outcomes of the current study were number of days at home the last 90 days of life and the proportion of home as place of death.

Secondary outcomes were number of days at hospital and nursing home, and hospital admissions during the last 90 days of life, number of fulfilled palliative plans, and number of days from first ACP conversation in primary health care to death and participants in the ACP conversations.

### Data collection

A data extract from the Norwegian Cause-of-death Register (Norwegian Institute of Public Health 12.12.18, project number 18-0503) gave an overview of number of patients per year who died from or with a cancer diagnosis in the nine municipalities.

A review of contact registration from the local hospital trust EPJ was used to identify palliative cancer patients who had contact with the local hospital, cancer outpatient clinic or hospital-based PC team and lived and died in one of the nine municipalities between September 2018 and August 2020. Additional data were extracted from hospital trust or municipality EPJ. Data included gender and age, place of death, number of hospital admissions and total number of hospital or nursing home days. Whole day stays at outpatient clinics like the oncology unit were not included as hospital stays. Information from community cancer nurses and documentation in municipality EPJ was used to identify patients who had an ACP conversation. Participants in these conversations were either documented in EPJ or the community cancer nurse gave additional information.

Contact with the hospital-based PC team was defined by appropriate documentation of a direct dialogue with the patient, collected from hospital EPJ.

Number of days, the patient was not admitted to hospital or nursing home was counted as days at home.

### Statistics

Descriptive statistics were used to summarize gender, age, number of days at home, in nursing home or in hospital, place of death, hospital admissions, ACP conversations (with participants and location) and palliative plan in primary health care, days from first ACP conversation to death and contact with the hospital-based PC team.

An independent two-sided t-test was used to examine differences in age between the two groups. Comparison analyses between the groups according to gender and contact with the hospital-based PC team were assessed by Pearson chi square test.

The data set was not normally distributed. A Poisson regression analysis was used to predict the association of days at home the last 90 days (dependent variable) with ACP conversation in primary healthcare, contact with hospital-based PC team, gender and age (predictors). To analyse statistically significant associations with home as place of death, we used again a Poisson regression analysis with the same predictors. The risk ratio (RR) gave additional information about the effect of ACP conversation on dying at home.

In all cases, *p* values < 0.05 were considered statistically significant. All statistical analysis was performed by using IBM SPSS Statistics version 27 (Statistical Product and Service Solutions).

### Ethics

The study has been performed in accordance with the relevant guidelines and regulations from the Declaration of Helsinki and was approved by Regional Committees for Medical and Health Research Ethics (REC – central; ID 119425 / 2020) and the Cancer Department at Møre and Romsdal Hospital Trust. REC-central granted a waiver of consent under the condition that data collection should be limited.

## Results

Data from the Norwegian Cause-of-death Register for the years 2008 to 2017 showed an expected average number of 350 patients dying from or with a cancer diagnosis in the nine municipalities in the Romsdal region over any two year period. This number was used to make sure we captured the majority of all cancer deaths in the time period of the study.

We identified 250 palliative cancer patients who had contact with the local hospital, cancer outpatient clinic or hospital-based PC team and died between September 2018 and August 2020. Among 125 (50 %) of these, we could verify a documented ACP conversation in primary health care setting.

Among the 250 patients, 148 were males and 102 females. The average age was 73.1 years (SD 11.9). Most of the patients, 222 (89%), had contact with the hospital-based PC team; 123 (98%) in the group with an ACP conversation in primary health care and 99 (79%) in the group without.

There were no significant differences between patients with or without an ACP conversation according to gender, *X*^2^(1, n = 250) = 1.06, *p* = 0.30, and age, t (248) = 0.52, *p* = 0.59, 95% CI (-2.2 – 3.8) (Table 1).

**Table 1:** Characteristics

	ACP conversation group (***N***=125)		Controls (N=125)		
					***p***-value
	(M*)	(SD*)	(M*)	(SD*)	
Age in years^a^	72,7	10,8	73,5	12,9	0.59
**Gender**^b^					0.3
male	78	62,4 %	70	56,0 %	
female	47	37,6 %	55	44,0 %	

*M = Mean, *SD* = Standard deviation

^a^Independent two-sided t-test for differences in age between the groups (t (248) = 0.52)

^b^Pearson chi square test for differences in gender between the groups (X^2^(1, *n* = 250) = 1.06)

### Primary outcomes

#### Days at home last 90 days of life

During the last 90 days of life the 125 patients who had an ACP conversation in primary health care were mean 9.8 more days at home than patients without this conversation; mean=65.8 days, SD=23.6 versus mean=56.0 days, SD=24.1, 95% CI of the difference (3.8 – 15.7) (Table 2). The Poisson regression analysis showed that three of four variables significantly predicted days at home the last 90 days of life. More days at home had patients who had an ACP conversation in primary health care (Wald *X*^2^ (1, n = 250) = 85,06; *p* < .001), males (Wald *X*^2^ (1, n = 250) = 22,98; *p* < .001) or patients with lower age (Wald *X*^2^ (1, n = 250) = 74,45; *p* < .001). No significant association was seen according to contact with the hospital-based PC team (Wald *X*^2^ (1, n = 250) = 0,79; *p* = .397).

**Table 2:** Place of care the last 90 days of life

	ACP conversation group (***N***=125)		Controls (***N***=125)		***p***-value
	(M*)	(SD*)	(M*)	(SD*)	
**Place of care (days)**					
home^c^	65,8	23,6	56,0	24,1	<0.001
nursing home	12,2	21,7	16,7	25,8	
hospital	12,0	11,6	17,3	14,8	
**Place of death**					
home^d^	53	42,4 %	12	9,6 %	<0.001
nursing home	54	43,2 %	57	45,6 %	
hospital	18	14,4 %	56	44,8 %	

*M = Mean, *SD* = Standard deviation

^c^Poisson regression analysis, dependent variable = days at home last 90 days of life, predictor = ACP conversation in primary health care (Wald X^2^ (1, *n* = 250) = 85,06)

^d^Poisson regression analysis, dependent variable = home as place of death, predictor = ACP conversation in primary health care (Wald X^2^ (1, n = 250) = 20,64)

#### Home as place of death

**Fig. 1:**
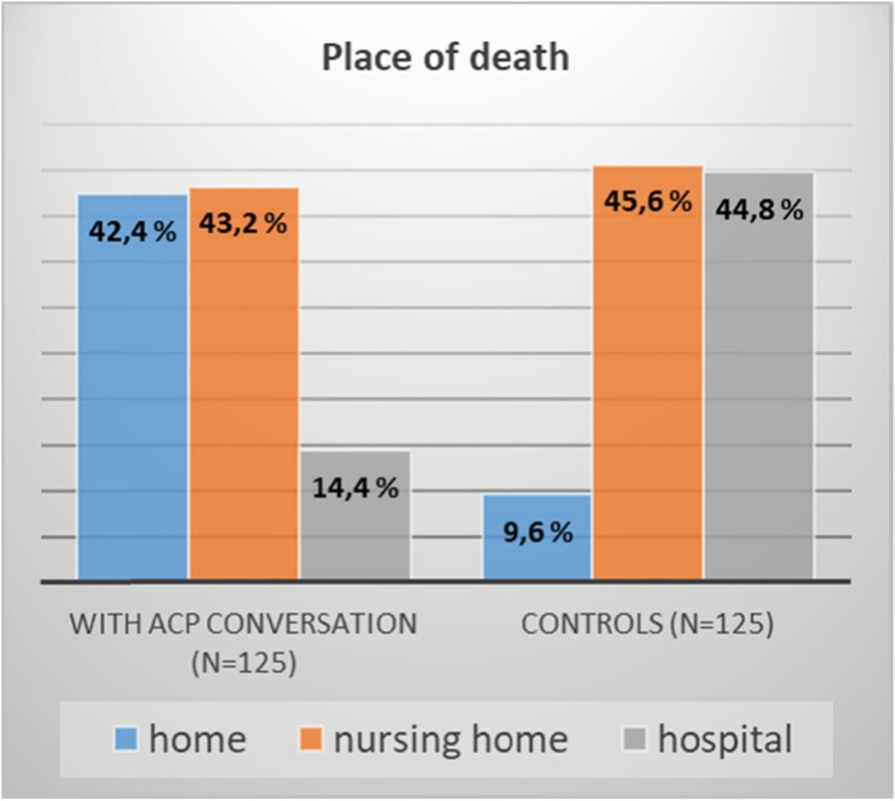
Place of death with and without ACP conversation in primary health care, *p* < 0.001 for death at home

Among the 125 cancer patients with an ACP conversation in primary health care, 53 (42,4%) died at home. In the group without an ACP conversation 12 (9,6%) died at home (FIG. 1).

The Poisson regression analysis showed that patients who had an ACP conversation in primary health care where significantly more likely to die at home (Wald *X*^2^ (1, n = 250) = 20,64; *p* < .001) with a four times higher probability (RR = 4.5; 95 % CI (2,36 - 8,67)). Contact with the hospital-based PC team (Wald *X*^2^ (1, n = 250) = 0,15; *p* = .695), gender (Wald *X*^2^ (1, n = 250) = 0,25; *p* = .874) or age (Wald *X*^2^ (1, n = 250) = 0,83; *p* = .361) had no predictive effect on home as place of death.

#### Secondary outcomes

During the last 90 days of life the 125 patients who had an ACP conversation in primary health care were mean 5.3 days less in hospital (mean=12.0, SD=11.6 versus mean=17.3, SD=14.8, 95% CI of the difference (2.0 – 8.6)) and 4.5 days less in nursing home (mean=12.2, SD=21.7 versus mean=16.7, SD=25.8, 95% CI (-1.5 – 10.4)) (Table 2).

Among the 125 cancer patients with an ACP conversation in primary health care, 54 (43,2%) died in nursing home and 18 (14,4%) in hospital. In the group without an ACP conversation, 57 (45,6%) died in nursing home and 56 (44,8%) in hospital (FIG. 1).

The number of hospital admissions during the last 90 days of life was similar in the two groups (mean=2.06, SD=1.56 versus mean=2.02, SD=1.34, t (248) = -0.2, p = .83, 95% CI (-0.4 - 0.3)).

The first **ACP conversation in primary health care** was mean 114.4 days (SD 131.9) before death of the patient (Table 3). Most of the conversations took place at the patient’s homes (84%), but also in nursing home (9%), at the GPs office (6%), or in hospital (1%). Participants in the ACP conversation were patients (99%), relatives (89%), community cancer nurses (81%), GPs (66%), home-care nurses (49%) and hospital-based PC team members (30%). Fifteen percent of the ACP conversations took place without a physician present.

**Table 3:** Secondary outcomes

	ACP conversation group (***N***=125)		Controls (N=125)		***p***-value
**Hospital admissions last 90 days**^e^					0.83
	(M*)	(SD*)	(M*)	(SD*)	
	2,1	1,6	2,0	1,3	
**First ACP conversation in primary health care**					
	(M*)	(SD*)			
Days before death	114,4	131,9			
**Contact with hospital-based PC team**^f^					<0.001
yes	123	98,4 %	99	79,2 %	
no	2	1,6 %	26	20,8 %	
**First contact with hospital-based PC team**^g^					0.001
	(M*)	(SD*)	(M*)	(SD*)	
Days before death	196,2	274,7	91,1	186,0	

*M = Mean, *SD* = Standard deviation

^e^Independent two-sided t-test for differences in hospital admissions between the groups (t (248) = -0.2)

^f^Pearson chi square test for differences in contact with hospital-based PC team between the groups (X^2^ (1, n = 250) = 23.2)

^g^Independent two-sided t-test for differences in first contact with hospital-based PC team between the groups (t (220) = -3.25)

Seventeen patients (13,6%) had an ACP conversation but no written palliative plan in primary health care. Twelve of these patients died shortly after the conversation, while five patients did not want to have a palliative plan.

Patients who had **contact with the hospital-based PC team** had significantly more often an ACP conversation in primary health care *X*^2^ (1, *n* = 250) = 23.2, *p* < 0.001 (Table 3).

The first contact with the hospital-based PC team was earlier before death for those who later had an ACP conversation in primary health care (mean 196.2 days, SD 274.7 compared to 91.1 days, SD 186.0 when there was no such conversation, t (220) = -3.25, *p* = 0.001, 95% CI (-168.81 - -41.46)) (Table 3).

## Discussion

This retrospective observational cohort study demonstrated a significant association between ACP conversations made in primary health care and more days at home at EoL for palliative cancer patients, compared to the control group. Additionally, we observed a reduction of days of hospitalization for patients with an ACP conversation and a reduction of days in nursing home. The patients with ACP conversations also more frequently died at home (42.4% versus 9.6% for the control group). Nursing home as place of death was similar in the two groups. Patients without an ACP conversation in primary health care more often died in hospital.

The study underlines our hypothesis that ACP conversations in primary health care is a method to support the wish of many patients to stay at home as long as possible and to get the opportunity to die at home. Cancer patients can reduce time away from loved ones at home.

GPs and community nurses are often the health care providers that patients and families rely on when exploring their values and preferences [21]. The preference to die at home is often reported as a goal of care [22]. This motivated us to start implementing ACP conversations in primary health care, with support from specialist PC.

The Dying Well in Europe study showed a high variability of rates of death at home in cancer patients across European countries with the lowest number in Norway (12.8%) [15]. Kjellstadli *et al.* showed latest in 2012 and 2013 that there were few home deaths in Norway and that even fewer people than anticipated have a potentially planned home death [16]. GPs and primary health care seem to play an essential role in enabling people to die at home [23]. There have been growing calls to keep GPs engaged in EoL care and to build capacity in providing a palliative approach to care in the primary care setting [24, 25]. It has been shown that where GPs were actively involved with home visits, ACP conversations and shared documentation of conclusions in a palliative plan, there was higher likelihood of home death for cancer patients [26]. The network of factors that influence where patients with cancer die is complicated and dependent on the illness, the individual, and the environment [27]. The results of our study support the importance of ACP conversations in primary health care setting, involving patient, family, community nurses and GP, to enable dying at home. Gender or age were no associated factors for home as place of death.

Continuity of care also seems to be an important factor in palliative home care [28]. Constantini *et al.* showed that ACP and a palliative home care team could decrease the number of cancer deaths in acute-care facilities [29]. Palliative home care has been shown to reduce time spent in hospital [29, 30] and increased time at home [31] in several studies using a time frame between 6 months and 30 days before death. Stein *et al.* showed that an intervention, consisting of an informational pamphlet and discussion, was associated with less likelihood of death in hospital [32]. Palliative home care can be offered by primary or specialist health care. Although hospital-initiated ACP by a patient's clinical health care team is feasible [33, 34], our data showed that contact with the hospital-based PC team didn’t have a significant association on days spent at home at the EoL. We found that patients with an ACP conversation in primary health care spent more time at home and less time in hospital during their last 90 days. The significant associations in the current study between more days at home and having lower age or being male is interesting but rarely discussed in literature. Lower age might influence patients not to choose nursing home as an alternative care facility. Partners of younger patients and especially female caregivers might have more capacity and competence as family caregivers. Men may rely on the care competence of their spouses / partners and women might be more comfortable with taking care for the husband / partner in home care setting in EoL. As described under methods, there is no hospice in Møre and Romsdal.

The role of the oncologist have been shown to be crucial for early integration of PC in oncology practice [35], and GPs appear to need encouragement for the early integration of ACP and PC for patients with advanced cancer [36]. In the current study, the hospital-based PC team supported primary health care in initiating ACP conversations. Early referral to hospital-based PC led more often to ACP conversations in primary health care, where GPs were involved. We suggest that a key factor to enable more time at home for advanced cancer patients is giving primary health care the necessary support on an individual patient level and on system level. Interventions at community level with training and support from expert teams in order to change the current care profile to a more outpatient care may allow a lower consumption of resources and longer care at home [37]. A joint responsibility between primary health care and specialist health care can be key component in planning and coordination of supportive-care domiciliary services [38].

Proactive identification of patients approaching EoL is likely to improve all aspects of care, including planning and communicating about EoL care [3]. Home care nurses have been shown to have a leading role in balancing the demands and the satisfaction when caring for someone dying at home [20]. Monitoring at risk people for the development of PC needs and providing advice and support for people with those needs is a part of normal nursing duties [21]. In the current study most often the community cancer nurses initiated the necessary communication around organizing the ACP conversation, where the GP but sometimes also a palliative care physician attended. Fifteen percent of the ACP conversations took part without a physician. In other countries community nurses play a similar role in initiating the conversations, but a more leading role in conducting these conversations on individual [39] and community level [40]. Timing to initiate an ACP conversation is variable and depends on experiences from health care providers. In a qualitative study, nurses identified that their decision to introduce an ACP discussion was influenced by an assessment of the patient's readiness to discuss the topic, their physical condition, and the nurse's relationship with the patient and family [41]. In our study, the first ACP conversations took place mean 114 days before death of the patient. We have no further information about the criteria primary health care professionals used to decide if and when they would offer an ACP conversation.

The data of the current retrospective study suggest that there is an association between ACP conversations in primary health care and more time at home and home as place of death. We believe that a possible explanation for this association might be a structured assessment and documentation of the patient’s goals of care within an active communication process in primary health care. Miccinesi *et al.* showed that most patients expressed their will to receive information on the disease process and/or the treatments proposed and they were willing to talk about what is important at the EoL, when they were asked [42]. ACP conversations initiated by specialist health care occurred mostly in late-stage cancer, with providers other than oncologists [43, 44]. GPs experience difficulties in initiating ACP if patients are being treated in the hospital [45]. It has been shown that the majority of the patients would prefer that such discourse take place with their primary medical provider [2]. Target-oriented communication between primary and specialist health care could facilitate communication between GPs and patients/families [46]. When compared to usual care, hospital-based specialist PC may offer increasing chances of patients dying in their preferred place (measured by home death) [8]. PC delivered by PC specialists without access to community-based PC means that PC will only be available for a minority of patients [47].

### Strengths and limitations

The study demonstrated associations between ACP conversations in primary health care and days spent in home or hospital in the last 90 days of life and home as place of death. The use of a retrospective cohort design allowed us to look at associations rather than a causative relationship.

Primary health care providers were responsible for offering, organizing, and conducting ACP conversations with patients. We have no further information about their selection criteria. While local clinical practices were similar in the participating communities, self-selection effects of the primary health care providers might have influenced the observed findings. It is possible that patients got an offer for an ACP conversation but declined. We didn’t gather data about frequency of ACP conversations for each patient, one documented ACP conversation in primary health care was enough to have the patient in the group with ACP conversation. Time duration of the ACP conversations was not documented in municipality EPJ. Conversations with patients as part of usual practice were possible but they happened without planning, organization and conclusive structured documentation and were not interpreted as ACP. Patients were identified independently of which cancer diagnosis they had.

The sample was drawn in a small local cancer population, which limits its generalizability to other populations, organizational systems, and communities. Only cancer patients with contact to specialist health care service were identified and included, covering probably 70% of all deaths from or with a cancer diagnosis in this region during the study period. We only had access to limited demographic and medical data due to not actively consenting patients for this study (ethically approved consent waiver). We did not analyse emergency department visits.

After the implementation of ACP conversations between January and June 2018, the cancer nurses in the communities gathered information about patients who got an ACP conversation and a summarizing palliative plan. This information was verified through corresponding documentation in community EPJ. We are confident that, for the cohort of patients in this study, we were able to identify all patients with documented ACP conversations in primary health care. Choosing the last 90 days of life according to place of care seems reliable as most ACP happened mean 114 days before death.

### Implications and further work

There is a tendency, even in today’s society, to avoid talking about dying. ACP conversations are currently regarded as very important for future health care systems, where death should be seen as a natural part of life. Clinical guidelines do recommend ACP for all patients with a chronic life-limiting illness like non-curable cancer [48]. An actual governmental statement in Norway calls for more openness about death and dying and claims that more people should be able to choose to stay longer at home and to die at home [49]. Our study supports that the necessary initiation of ACP and communication with the patient and family should be conducted in primary health care setting.

Ongoing follow up studies with a prospective design will be of high value to evaluate patient’s preferred place of care and death, and the generalizability of primary palliative care programs across different communities. A prospective study is needed to be able to determine the relationships of ACP conversation use in primary health care, time at home and place of death.

The GP together with community cancer nurses and home care nurses should be key caregivers within ACP if patient relationship is established and effective. The hospital-based PC team can identify PC needs early and actively support initiation of an ACP conversation in primary health care. This gives the opportunity to manage upcoming needs of the dying person and his / her family and makes home setting, when preferred by the person, the best place to be the last 3 months of life.

## Conclusions

In this retrospective observational cohort study, an ACP conversation in primary health care was significantly associated with more days at home during the last 90 days of life for palliative cancer patients, and more frequently home as place of death. On top of satisfied patients, which can spend more time and die at home, this approach saves the hospital system a lot of money. Contact with the hospital-based PC team supported community health care providers to initiate communication about ACP. A future strategy for the transition of palliative cancer patients to a home-based program could be a joint responsibility, where specialist health care and a hospital-based program prepares patients and relatives and supports GPs and home care nurses on behalf in EoL care at home.

The findings provide important information on our way towards a home model within PC by integrating oncology palliative care into the framework of primary health care disease management. The dataset supporting the conclusions of this article and a detailed information about these data is included within the article as additional files 1 and 2.

## Supplementary Information

**Additional file 1.**

**Additional file 2.**

## Abbreviations

ACP: Advance care planning
EoL: End-of-Life
EPJ: Electronic Patient Journal
GP: General Practitioner
PC: Palliative care
RR: Relative Risk
