# Supplementary material for: Cancer patients spend more time at home and more often die at home with advance care planning conversations in primary health care: a retrospective observational cohort study
Source: BMC Palliat Care. 2022 May 2;21:61. doi: 10.1186/s12904-022-00952-1 (PMC9063101; doi:10.1186/s12904-022-00952-1)
Supplement: Supplementary file 2 — Additional file 2. [file 12904_2022_952_MOESM2_ESM.docx]

This README file was generated on 2021-11-18 by Bardo Driller.

Last updated: 2021-11-22.

-------------------

GENERAL INFORMATION

-------------------

// Title of Dataset: ACP conversations with cancer patients in primary health care

// DOI:

// Contact Information

// Name: Bardo Driller

// Institution: Møre og Romsdal Hospital trust, Oncology Department

// Email: bardo.driller@helse-mr.no

// ORCID: https://orcid.org/0000-0003-3611-5080

// Contributors: See metadata field Contributor.

// Kind of data: See metadata field Kind of Data.

// Date of data collection/generation: See metadata field Date of Collection.

// Geographic location: See metadata section Geographic Coverage.

// Funding sources: No funding.

// Description of dataset:

The dataset describes information about whether patients dying from or with cancer in our region between September 2018 and August 2020 had an Advance care planning (ACP) conversation in primary health care or not. We wanted to see at associations between ACP conversation and time at home and place of death.

All patients were registered with age, gender and date of death. Additionally we registered if, and if yes, when patients had contact to the hospital based palliative care (PC) team. For patients with an ACP conversation we documented the date of the first conversation and date of finishing the first Palliative plan.

All patients had data according to how many days they spent in home, nursing home or hospital and how often they were admitted to hospital during their last 90 days of life. Additionally patients place of death was verified.

--------------------------

METHODOLOGICAL INFORMATION

--------------------------

Setting

In 2018, Møre and Romsdal county in North-western Norway started providing ACP conversations and a structured palliative plan in primary health care to individuals with life-limiting illnesses like non-curable cancer.

Study design

The current retrospective observational cohort study evaluated outcomes from cancer patients enrolled in the ACP conversation program, compared with a control group that did not receive the ACP conversation. The study was conducted in nine municipalities in the Romsdal region with 65,000 inhabitants.

Subjects

The study included cancer patients that 1) lived in one of the nine municipalities in the Romsdal region, 2) had contact with the local hospital, cancer outpatient clinic or hospital-based PC team, and 3) died between September 2018 and August 2020.

After implementation in 2018, patients relevant for ACP conversations and a conclusive palliative plan started a communication process in primary health care. During this process patients and/or their relatives had organized ACP conversations together with health care providers to consider patient’s wishes and preferences towards future health care. The primary health care provider had the necessary information about medical status and prognosis of the patient. All participants had information about the intention of the ACP conversation. Confirmed conclusions from the ACP conversation was documented as a palliative plan in the electronic patient journal (EPJ). With permission from the patient, the plan was electronically available for all future health care providers in the region. The palliative plan was reassessed on demand when the patient’s medical condition changed, favourably based on a new ACP conversation.

Control group

The control group consisted of cancer patients who did not have an ACP conversation and a palliative plan in the primary health care setting.

Primary and secondary outcomes

Primary outcomes of the current study were number of days at home, in nursing home or in hospital the last 90 days of life and place of death.

Secondary outcomes were number of hospital admissions during the last 90 days of life, number of fulfilled palliative plans, and number of days from first ACP conversation in primary health care to death and participants in the ACP conversations.

Data collection

A data extract from the Norwegian Cause-of-death Register (Norwegian Institute of Public Health 12.12.18, project number 18-0503) gave an overview of number of patients per year who died from or with a cancer diagnosis in the nine municipalities.

A review of contact registration from the local hospital trust EPJ was used to identify palliative cancer patients that had contact with the local hospital, cancer outpatient clinic or hospital-based PC team and lived and died in one of the nine municipalities between September 2018 and August 2020. Additional data was extracted from hospital trust or municipality EPJ. Data included gender and age, place of death, number of hospital admissions and total number of hospital or nursing home days. Whole day stays at outpatient clinics like the oncology unit were not included as hospital stays. Documentation in municipality EPJ was used to identify patients who had an ACP conversation. Participants in these conversations were either documented in EPJ or the community cancer nurse gave additional information.

Contact with the hospital-based PC team was defined by appropriate documentation of a direct dialogue with the patient, collected from hospital EPJ.

Number of days, the patient was not admitted to hospital or nursing home was counted as days at home.

17 patients had an ACP conversation but did not get a palliative plan, they got no number in their column. From these 17 patients 12 died too fast to could get a signed Palliative plan and 5 patients did not wat to have a Palliative plan. Analyses between patients with or without a Palliative plan in primary health care were done with 108 patients in group with ACP conversations and a Palliative plan and 125 controls.

Specialist contribution means that the specialist PC team wrote a note to support initiating or updating the palliative plan in primary health care. A direct patient contact was a requirement to such note.

// Facility-, instrument- or software-specific information needed to interpret the data:

We used Microsoft Excel 2016 to gather the data and calculate for example days from first ACP conversation to death.

// Describe any quality-assurance procedures performed on the data:

During collection of data we verified that date of death was in between the two years period and we used a sum function to verify to look at exact the last 90 days of life.

--------------------

DATA & FILE OVERVIEW

--------------------

// File List:

ACPConversationBD2021.txt is the only file with data about ACP conversations in primary health care. This is the first public version of this data set.

Additionally the ReadMe file ACPConBDReadme.txt describes the data set.

-----------------------------------------

DATA-SPECIFIC INFORMATION FOR: ACPConversationBD2021.txt

-----------------------------------------

<Repeat this section for each dataset, folder or file, as appropriate. Recurring items may also be explained in a common initial section.>

<For TABULAR data, provide a data dictionary/code book containing the following information:>

// Variable/Column List:

ID; age; gender 0=male 1=female; date palliative plan in primary health care; palliative plan in primary health care 0=no 1=yes; date ACP conversation in primary health care; ACP conversation in primary health care 0=no 1=yes ; place of ACP conversation in primary health care 0=home 1=GP office 2=nursing home 3=hospital; date of death; place of death 0=home 1=nursing home 2=hospital; days at home the last 90 days; days in nursing home the last 90 days; days in hospital the last 90 days; hospital admissions the last 90 days; date first kontakt to spesialist palliative care team; date first spesialist contribution to palliative plan; date last spesialist contribution to palliative plan; days from first palliative plan in primary health care to death; days from ACP conversation in primary health care to death; days from first specialist contribution to palliative plan to death; days from last specialist contribution to palliative plan to death; place of death 1=primary health care 2=specialist health care

<List variable/column name(s), description(s), unit(s) of measurement, decimal separator (comma or point), value labels, and source(s) as appropriate for each.>

// Missing data codes:

none

--------------------------

SHARING/ACCESS INFORMATION

--------------------------

<Whenever applicable, the following information should be registered in the metadata schema of DataverseNO. In the text below, remove fields that are not applicable, and leave the rest unchanged. >

// Licenses/Restrictions: See Terms tab.

// Links to publications that cite or use the data: See metadata field Related Publication.

// Links/relationships to related data sets: See metadata field Related Datasets.

// Data sources: See metadata field Data Sources.

// Recommended citation: See citation generated by repository.
